# Editorial: The inaugural Monash international health science and technology conference: pharmacology perspectives

**DOI:** 10.3389/fphar.2023.1208546

**Published:** 2023-05-09

**Authors:** Kooi Yeong Khaw, Michael R. Whittaker, Sanjaya Kuruppu, Kok Gan Chan, Siew Hua Gan, Bey Hing Goh

**Affiliations:** ^1^ School of Pharmacy, Monash University Malaysia, Selangor, Malaysia; ^2^ Drug Delivery, Disposition and Dynamics Theme, Monash Institute of Pharmaceutical Sciences, Monash University, Parkville, VIC, Australia; ^3^ Department of Biochemistry and Molecular Biology, Faculty of Medicine, Nursing and Health Sciences, Monash University, Clayton, VIC, Australia; ^4^ Division of Genetics and Molecular Biology, Institute of Biological Sciences, Faculty of Science, University of Malaya, Kuala Lumpur, Malaysia; ^5^ International Genome Centre, Jiangsu University, Zhenjiang, China; ^6^ College of Pharmaceutical Sciences, Zhejiang University, Zhejiang, China; ^7^ Biofunctional Molecule Exploratory (BMEX) Research Group, School of Pharmacy, Monash University Malaysia, Selangor, Malaysia

**Keywords:** cancer, phytochemistry, nanoparticles, *Polygonum odoratum*, Scutellaria barbata

The Research Topic aims to gather selected pharmacology-focused contributions to, *The Inaugural Monash International Health Science and Technology Conference* (MONASH INITIATE), with the theme “Inspiring Innovation via Multidisciplinary Collaboration,” held in the School of Pharmacy of the Monash University Malaysia on 16th–17th June, 2021. The conference attracted over 100 local and international attendees with participation from Malaysian and foreign universities, research institutions and industry partners.

The Research Topic compiles five high-quality review articles contributed by scientists in the field. The Research Topic of these publications revolve around cancer therapy, the traditional uses of medicinal plants, and the molecular mechanisms of phytochemicals in anticancer applications. These articles provide comprehensive overviews of the current research and knowledge in drug delivery and natural products-based drug discovery areas.

A scoping review article by Miatmoko et al., outlines the delivery of Ursolic acid by nano particles, namely, liposomes, nanospheres and polymeric micelles in cancer therapy. The authors highlighted that nanoparticles in particular are effective in improving relative survival rate; reducing tumor resistance, and improving tumor tissue histopathology. They are safe to be used preclinically and clinically, and it is worth noting that the use of liposome delivery of ursolic acid is in a phase 1 clinical trial.

The systematic review article by Arbain et al., discussed the distribution, traditional uses, phytochemicals and biological activities of 96 species of Alocasia. The genus is distributed across Asia, Southeast Asia and Australia. Phytochemicals from the genus of the plants can be categorized into lignan, saponin, alkaloids, and phenolic compounds. Despite demonstrating numerous biological activities, including antidiabetic, antinoceptive, antiinflammation effects, the author highlighted the compound alocasgenoside B from *A. cucullata* tuber was potently cytotoxic to the cancer cells *in-vitro*.

A systematic review by Khuayjarernpanishk et al., outlined the anticancer activities of *Polygonum odoratum* Lour. The review highlighted eight anticancer studies from the phytochemicals of *Polygonum odoratum* including Flavanoids, tannins, and saponins. A range of cancer cell lines was screened and their anticancer activity reported against oral, lung, breast, colon, liver, T lymphoblast, lymphoma, and leukemia cells. The mechanistic study was reported, and included the inhibition of Akt expression, phosphorylated Akt, mTOR, and phosphorylated mTOR. The authors also identified the gaps and limitations in the current research that warrants future study, including the discovery and identification of different classes of phytochemicals as well as informed *in-vivo* studies.

Meanwhile, Yang et al., focuses on the mechanism of action of *Scutellaria barbata* on hepatocellular carcinoma. A rather systematic multi-step investigation biodiscovery approach was utilized in this study. Initially, the active components of *Scutellaria barbat*a were identified using a database of traditional Chinese medicine compounds. Then, the potential targets of these active components were predicted using network pharmacology tools. Following that, the hepatocellular carcinoma related genes were collected from various databases, and the overlapping targets between *Scutellaria barbata* and hepatocellular carcinoma related genes were identified. Further analysis was conducted to explore the potential biological functions and pathways involved in the interactions between *Scutellaria barbata* and hepatocellular carcinoma. Eventually, the effect of *Scutellaria barbata*, a widely used medicinal herb in traditional Chinese medicine, was found to exert modulation of various signaling pathways, such as the PI3K/AKT, MAPK, and NF-κB pathways in hepatocellular carcinoma.

Finally, Liang et al., reviews current literature on the anticancer applications of phytochemicals in gastric cancer. It summarizes the effects of different phytochemicals, such as flavonoids, phenolic compounds, alkaloids, and terpenoids, on gastric cancer cells in laboratory settings and animal models. Various mechanism of actions associated with the anticancer effect were detailed, including inhibiting cancer cell proliferation, inducing apoptosis (programmed cell death) in cancer cells, suppressing angiogenesis (formation of new blood vessels that supply tumors), inhibiting metastasis (spread of cancer to other parts of the body), and modulating signaling pathways involved in cancer development and progression. The article articulates the potential synergistic effects of combining different phytochemicals or combining phytochemicals with conventional chemotherapy or radiotherapy in the treatment of gastric cancer. It also emphasized a discussion on safety and bioavailability of phytochemicals, and their potential use as adjuvants to conventional therapies in gastric cancer patients.

These publications provide valuable insights into the current research on cancer therapy which exploit the traditional uses of medicinal plants, nanoparticles, and the potential integration of network pharmacology methodology in identifying bioactive(s) and the elucidation of their molecular mechanism actions in cancer treatment. These publications contribute to the understanding of these fields and may serve as references for further research and development of novel cancer therapies. The editorial team would like to express gratitude to the review editors, reviewers, and authors for their contribution to this Research Topic. The editorial team is looking forward to having interested researchers participate in the forthcoming Monash INITIATE 2023 on 25th–26th October 2023 at Monash University Malaysia.

